# Efficacy and predictor of anti-TNFα agents in patients with intestinal Behçet's disease

**DOI:** 10.1186/s12876-022-02221-0

**Published:** 2022-03-28

**Authors:** Haruka Miyazaki, Daisuke Watanabe, Norihiro Okamoto, Eri Tokunaga, Yuna Ku, Haruka Takenaka, Namiko Hoshi, Makoto Ooi, Yuzo Kodama

**Affiliations:** 1 Division of Gastroenterology, Department of Internal Medicine, Kobe University Graduate School of Medicine, 7-5-1 Kusunoki-cho, Chuo-ku, Kobe, 650-0017 Japan; 2 Department of Gastroenterology, University of Michigan, 6520C MSRB I, SPC 5682, 1150 W Medical Center Dr, Ann Arbor, MI 48109 USA; 3 Division of Gastroenterology, National Hospital Organization Kobe Medical Center, Kobe, Japan

**Keywords:** Intestinal Behçet’s disease, Infliximab, Adalimumab, CRP

## Abstract

**Background:**

Behçet’s disease (BD) is a recurrent multisystem inflammatory disease. Anti-tumor necrosis factor (TNF) α agents have been used to treat patients with intestinal BD with severe disease activity or those who are resistant to conventional treatments; however, the long-term efficacy of anti-TNFα agents in intestinal BD remains unclear. In the present study, we investigated the clinical outcomes and predictors of discontinuation of anti-TNFα agents in patients with intestinal BD.

**Methods:**

We reviewed the medical records of patients with intestinal BD who received first-line anti-TNFα agents between January 2009 and June 2020. The primary outcome was the percentage of patients who continued anti-TNFα therapy for 48 weeks. Secondary outcomes included the percentage of patients who achieved marked improvement, complete remission, and mucosal healing, as well as predictors of discontinuation of anti-TNFα agents.

**Results:**

A total of 29 patients were included in the study. Twenty-two (75.9%) patients continued anti-TNFα therapy for 48 weeks. The percentage of patients who achieved marked improvement, complete remission, and mucosal healing at week 48 was 48.3%, 37.9%, and 48.3%, respectively. At week 96, 11 (37.9%) patients achieved marked improvement, complete remission, and mucosal healing. A higher C-reactive protein level (CRP; ≥ 1 mg/dL) at baseline was a predictor of discontinuation of anti-TNFα agents.

**Conclusions:**

The 48-week continuation rate of anti-TNFα agents was 75.9% in bio-naïve patients with intestinal BD. However, a higher baseline CRP level (≥ 1 mg/dL) was associated with discontinuation of anti-TNFα agents.

## Background

Behçet's disease (BD) is a chronic multisystem inflammatory disorder characterized by recurrent oral and genital ulcers and ocular and skin lesions [1]. In Japan, a BD diagnosis is reached by assessing a combination of clinical manifestations [2], as there are no disease-specific symptoms or laboratory tests for diagnosis.

Patients are diagnosed with intestinal BD if gastrointestinal symptoms are present, and typical ulcerative lesions are documented by employing objective measures. In BD, typical intestinal lesions are usually characterized by discrete ulcers with a punched-out appearance, located most commonly on the ileocecal valve [3].

The incidence of gastrointestinal manifestations is reportedly 3–25% in patients with BD [1]. In addition, patients with intestinal BD present a high risk for intestinal perforation and severe intestinal bleeding [4]; therefore, disease activity should be strictly controlled. Moreover, optimal treatment strategies are yet to be established, as intestinal BD is extremely rare, and the choice of treatment modality depends on disease severity [5, 6]. Among the available treatments for intestinal BD, 5-aminosalicylic acid (5-ASA), systemic corticosteroids, and immunosuppressive agents such as thiopurines are known to be useful in clinical settings [7]. 5-ASA is used in mild cases, and the cumulative relapse rates of intestinal BD at 1 and 5 years after remission were 8.1% and 31.2%, respectively [8]. Although there have been no prospective studies demonstrating the clinical efficacy of systemic corticosteroids in intestinal BD, they are often empirically used to induce clinical remission in patients with moderate to severe disease activities. Azathioprine (AZA) and 6-mercaptopurine (6-MP) are also widely used in patients who are dependent and refractory to corticosteroids. Jung et al. have reported that the cumulative relapse rates at 1 and 5 years after remission were 5.8% and 51.7%, respectively [9]. Despite the availability of these treatments, some patients experience treatment failure or require surgery [10].

Notably, anti-tumor necrosis factor-α (anti-TNFα) agents are now expected to be promising treatment options, with accumulating evidence revealing the therapeutic efficacy of anti-TNFα agents such as infliximab (IFX) and adalimumab (ADA) [6, 11, 12]. IFX therapy has shown a complete response rate of 61% at 54 weeks post-induction [13], while that of ADA was 60% at 52 weeks post-induction [14, 15]. In Japan, IFX and ADA have recently been approved for treating patients with intestinal BD exhibiting severe disease or resistance to existing treatment. Accordingly, anti-TNFα agents are expected to gain momentum in the management of intestinal BD [6]. However, data regarding the long-term outcomes of anti-TNFα therapy remain scarce.

In the present study, we retrospectively evaluated continuation rate of anti-TNFα in intestinal BD and factors predictive of sustained response.

## Methods

### Patients

Among 51 patients with intestinal BD, we reviewed the medical records of all patients who received first-line anti-TNFα agents at Kobe University Hospital from January 2009 to June 2020. The diagnosis of intestinal BD was based on the Japanese diagnostic criteria for intestinal BD [6]. We excluded patients with any evidence of other gastrointestinal diseases, such as Crohn's disease (CD), intestinal tuberculosis, or ischemic colitis, during the follow-up period.

### Drug administration

IFX or ADA was administered according to the standard Japanese administration protocol. IFX (5 mg/kg) was intravenously administered at weeks 0, 2, and 6, and then every 8 weeks. ADA was subcutaneously administered at 160 mg at week 0, followed by 80 mg at week 2, followed by 40 mg every other week. Concomitant therapies, such as corticosteroids and immunosuppressants, are permitted at stable dosages during anti-TNFα therapy. Upon observation of clinical improvement, corticosteroids were gradually reduced and discontinued.

### Data collection and definition

**Fig. 1:**
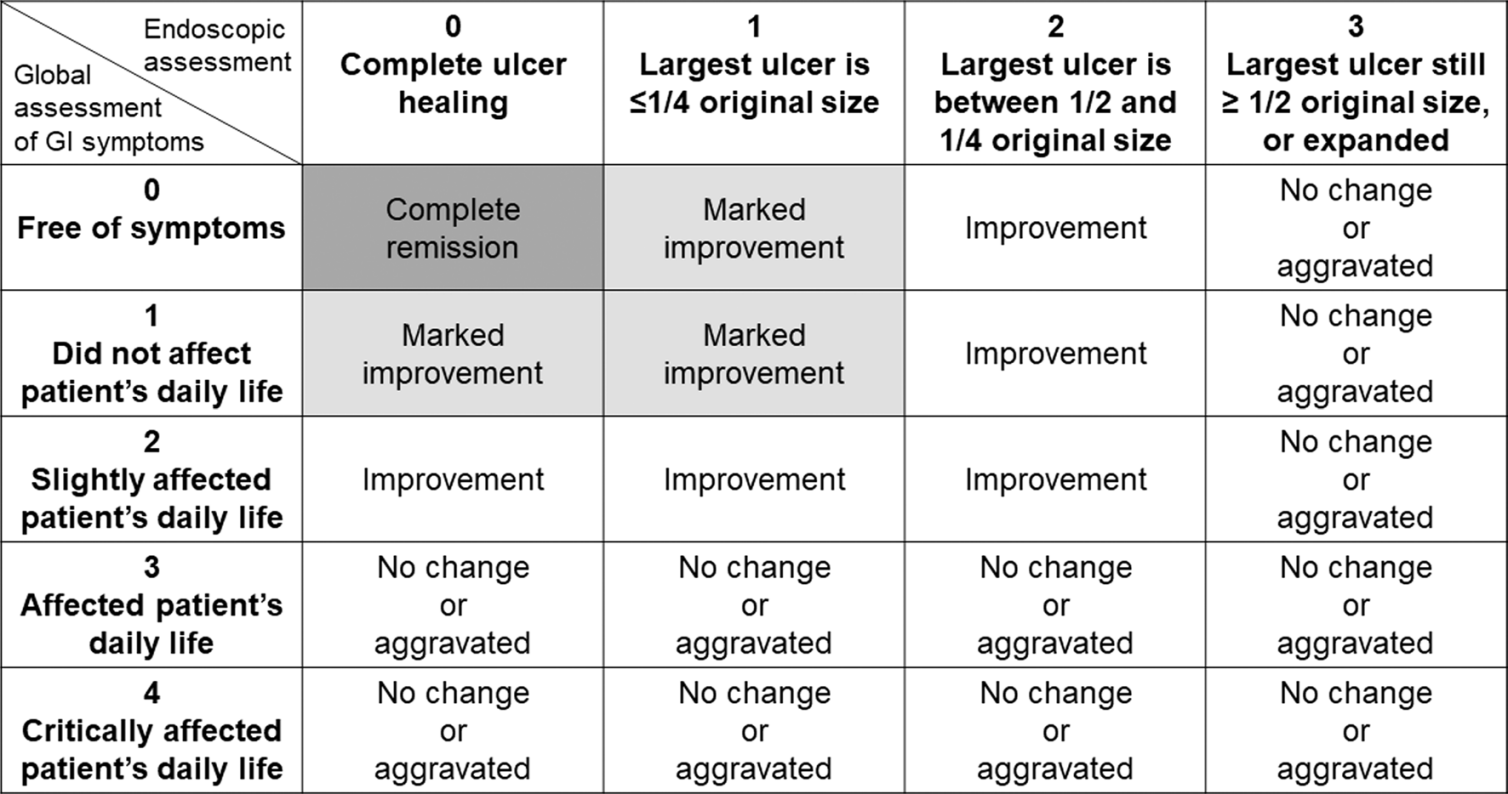
The composite disease activity index for global GI symptom and endoscopic assessment scores. A combination of scores is used to characterize disease activity after medical treatment

We collected information regarding sex, age at disease onset, body weight, social history (smoking and alcohol drinking), type of anti-TNFα agent administered, age at initial administration of the anti-TNFα agent, disease duration before initiating the anti-TNFα agent, presence of clinical manifestations, positivity for human leukocyte antigen (HLA)-B51 or HLA-A26 (HLA types highly associated with BD), concomitant medication administered with the anti-TNFα agent, history of major abdominal surgery (e.g., colectomy), and adverse events (AEs). First, we investigated the efficacy and safety by evaluating the continuation of anti-TNFα agents. Patients who continued anti-TNFα agents according to the administration protocol were defined as the continuation group (CG). The remaining patients were considered not to have continued therapy, as they deviated from the administration protocol (increasing dose, shortening interval, switching of anti-TNFα agent, or adding other treatments). These patients were defined as the discontinuation group (DG). Next, we used the composite disease activity index to comprehensively evaluate the efficacy of the anti-TNFα agent (Fig. 1), used in Japanese clinical trial for the obtainment of pharmaceutical approval of ADA for refractory intestinal BD (clinical trial number: NCT01243671) [14]. Global gastrointestinal (GI) symptom scores (ranging from 0 to 4 at the time of each visit) were assessed before the initial administration and 48 and 96 weeks after the initial administration of anti-TNFα agents. Endoscopic findings were evaluated by comparing changes in ulcer size with the original ulcer size before initiating anti-TNFα administration; score 0 was defined as complete ulcer healing, score 1 was defined as an ulcer size ≤ 1/4 the size of the largest ulcer when compared with the original ulcer size, score 2 was defined as ulcer size between 1/2 and 1/4 the size of the largest ulcer, and a score of 3 was defined as an ulcer size ≥ 1/2 of the largest ulcer.

Global drug responses were assessed by dividing the observed responses into four categories: no change/aggravated, improvement, marked improvement (MI), and complete remission (CR), using the composite disease activity index at the indicated time points, consisting of global GI symptom scores and endoscopic assessments. MI was defined as a global GI symptom score and endoscopic assessment score of ≤ 1, while CR was defined as a global GI symptom score and an endoscopic score of 0. In addition to the global drug response, mucosal healing (MH) was defined separately when the endoscopic score was 0.

### Primary and secondary endpoints of our study

In the present study, the primary outcome was the continuation rate of anti-TNFα therapy at week 48. The secondary outcomes included the percentage of patients who achieved MI, CR, and MH at weeks 48 and 96, the predictors of discontinuation of anti-TNFα therapy, and all AEs through week 96.

### Statistical analysis

All data are summarized and presented as mean ± standard deviation (SD) for continuous variables. Categorical data are expressed as the number of patients plus the percentage. Comparisons of groups were performed using the Student's *t-test* for unpaired data in a two-group comparison. The Chi-square test with Fisher's correction was used to evaluate differences in categorical data, where needed. Statistical significance was set at a *p* value of ≤ 0.05. The Kaplan–Meier survival method was used to estimate the cumulative probability of continuing anti-TNFα therapy. Differences between curves were tested using the log-rank test. EZR software was used for the statistical analyses.

## Results

### Baseline characteristics of patients (Table 1)

A total of 29 patients with intestinal BD were included in this study. The baseline patient characteristics are shown in Table 1. Among the 29 patients, 14 (48.3%) were men. The average age at initiation of anti-TNFα therapy was 42 years (range 17–74 years), and the average disease duration before anti-TNFα therapy was 6 years (range 0–41 years). Regarding the type of anti-TNFα agent, 16 (55%) patients were treated with IFX and 13 (45%) were treated with ADA. Ocular lesions, skin lesions, oral ulcers, and genital ulcers were present in 9, 12, 28, and 10 patients, respectively. In terms of HLA serological typing (A/B), 21 patients (21/29, 72.4%) were investigated for HLA serological typing (A/B). We identified 5 (5/21, 23.8%) patients positive for HLA-B51 and three (3/21, 14.3%) positive for HLA-A26. Concomitant medications administered with anti-TNFα agents included systemic corticosteroids (20/29, 69.0%), 5-ASA (11/29, 37.9%), cyclosporine A (2/29, 6.9%), methotrexate (3/29, 10.3%), AZA/6-mercaptopurine (18/29, 62.1%), and colchicine (16/29, 55.2%). Four patients (13.8%) had a history of major abdominal surgery. The mean baseline global GI score was 1.69, while the mean baseline C-reactive protein (CRP) level was 1.81 mg/dL.

**Table 1:** Baseline characteristics of patients

	n = 29
Male sex (%)	14 (48.3)
Age at disease onset (mean ± SD, years old)	36 ± 14
Body weight (kg)	
Mean ± SD	51.7 ± 11.1
Range	32.0–78.0
Tobacco, nonsmoker (%)	22 (75.9)
Alcohol, nondrinker (%)	17 (58.7)
Age at initiation of anti-TNFα agent (mean ± SD, years old)	42 ± 14
Disease duration before anti-TNFα agent (mean ± SD, years)	6 ± 9
Type of anti-TNFα agent (IFX/ADA) administered	16/13
Major symptoms	
Ocular lesions (%)	9 (31.0)
Skin lesions (%)	12 (41.4)
Oral ulcers (%)	28 (96.6)
Genital ulcers (%)	10 (34.5)
Minor symptoms	
Arthritis (%)	8 (27.6)
Vascular involvement (%)	1 (3.4)
HLA-B51 positivity (n = 21, %)	5/21 (23.8)
HLA-A26 positivity (n = 21, %)	3/21 (14.3)
Concomitant medication administered with the anti-TNFα agent	–
Systemic corticosteroids (%)	20 (69.0)
≧ 20 mg corticosteroids (%)	9 (31.0)
5-Aminosalicylic acid (%)	11 (37.9)
CyA (%)	2 (6.9)
MTX (%)	3 (10.3)
Azathioprine/6-mercaptopurine (%)	18 (62.1)
Colchicine (%)	16 (55.2)
Previous major abdominal surgery (%)	4 (13.8)
baseline Global GI symptoms score (mean ± SD); score 0–4	1.69 ± 1.4
Baseline CRP level (mean ± SD, mg/dL)	1.81 ± 3.8

ADA, adalimumab; CRP, C-reactive protein; CyA, cyclosporine A; HLA, human leukocyte antigen; IFX, infliximab; MTX, methotrexate; SD, standard deviation; TNFα, tumor necrosis factor α

During the observation period, AEs occurred in 14 patients (48.3%). AEs included nasopharyngitis in 7 patients (24.1%), hepatic events in 2 patients (6.9%), dizziness in 2 patients (6.9%), urinary tract infection in 1 patient (3.4%), pulmonary infection in 1 patient (3.4%), influenza in 1 patient (3.4%), and headache in 1 patient (3.4%). Among these, two serious events were observed. One patient died of lung cancer, which developed one year after the initial IFX administration. The other patient experienced ileum perforation due to exacerbation of intestinal BD.

### Relation between failure of anti-TNFα agent and CRP levels (Table 2)

Next, we evaluated the continuation rate of anti-TNFα therapy. As shown in Table 2, 25 (86.2%) and 22 (75.9%) patients continued anti-TNFα therapy at weeks 8 and 48, respectively. We compared CRP levels at baseline in the CG and DG groups. At week 8, the mean CRP levels at baseline in the CG and DG were 1.16 ± 2.09 mg/dL and 5.93 ± 8.60 mg/dL, respectively (*p* = 0.017). At week 48, the mean CRP levels at baseline in the CG and DG were 0.84 ± 1.41 mg/dL and 4.86 ± 6.80 mg/dL, respectively (*p* = 0.006).

**Table 2:** Relationship between the continuation of anti-TNFα agent and CRP levels

	At week 8			At week 48		
	Continuation	Discontinuation	*p* value	Continuation	Discontinuation	*p* value
N (%)	25 (86.2)	4 (13.8)		22 (75.9)	7 (24.1)	
CRP level at baseline (mean ± SD, mg/dl)	1.16 ± 2.09	5.93 ± 8.60	0.017	0.84 ± 1.41	4.86 ± 6.80	0.006
CRP ≥ 1 mg/dL in the group (%)	9 (36.0)	2 (50.0)		6 (27.3)	5 (71.4)	

CRP, C-reactive protein; SD, standard deviation

Moreover, 9 (36.0%) patients in the CG had baseline CRP levels of 1 mg/dL or higher, whereas two (50.0%) patients in the DG showed similar levels. At week 48, 6 (27.3%) patients in the CG had baseline CRP levels of 1 mg/dL or higher when compared with 5 (71.4%) patients in the DG.

### Continuation rates and efficacy of anti-TNFα agents (Figs. 2, 3)

**Fig. 2:**
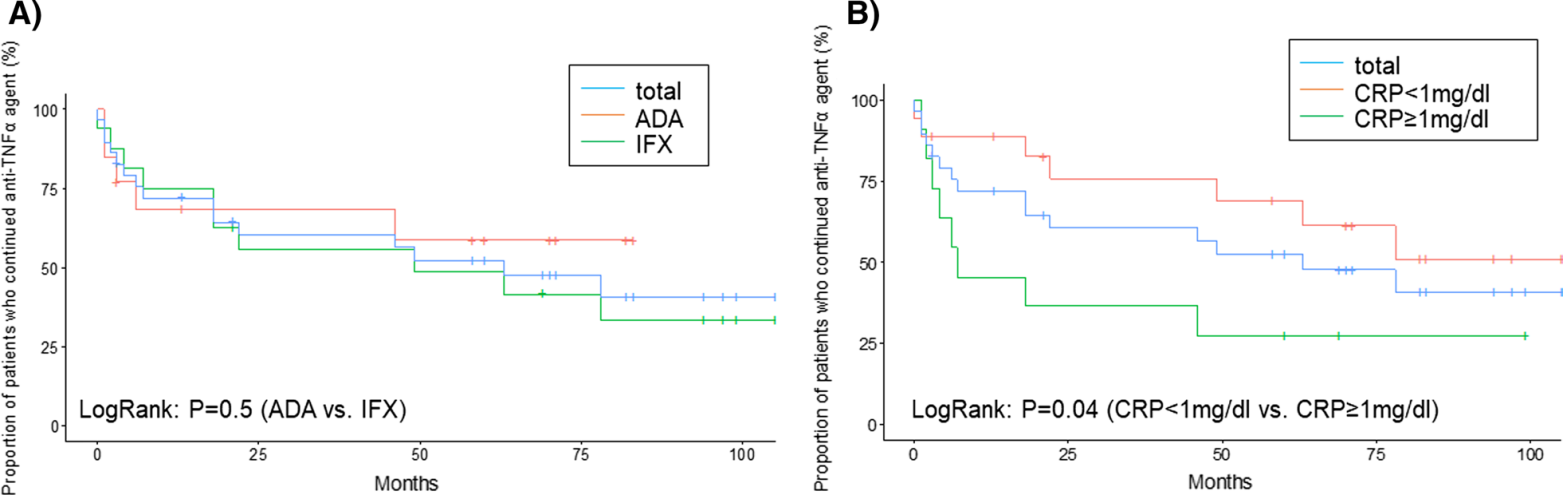
The cumulative continuation rate of anti-TNFα agents was calculated by using the Kaplan–Meier curve. A There is no significant difference between IFX users and ADA users. B A significant difference can be observed between patients with a baseline CRP level ≥ 1 mg/dL and those with a baseline CRP level < 1 mg/dL

We investigated the overall clinical course of patients with intestinal BD who received first-line anti-TNFα therapy (Fig. 2). The cumulative continuation rate of anti-TNFα agents was calculated using the Kaplan–Meier curve. Kaplan–Meier curves demonstrated that the cumulative continuation rates of anti-TNFα agents at weeks 8, 48, and 96 were 86.2%, 72.0%, and 60.4%, respectively. The median duration of continuation was 63 months.

Furthermore, we compared the cumulative continuation rates calculated by the Kaplan–Meier curve according to the type of anti-TNFα agent (IFX or ADA) and the difference in baseline CRP levels (CRP ≥ 1 mg/dL vs. CRP less than 1 mg/dL) using the log-rank test (Fig. 2). No significant difference in cumulative continuation rates was observed between the IFX and ADA groups (*p* = 0.5). In contrast, baseline CRP levels of 1 mg/dL or higher were associated with discontinuation of anti-TNFα agents (*p* = 0.04).

**Fig. 3:**
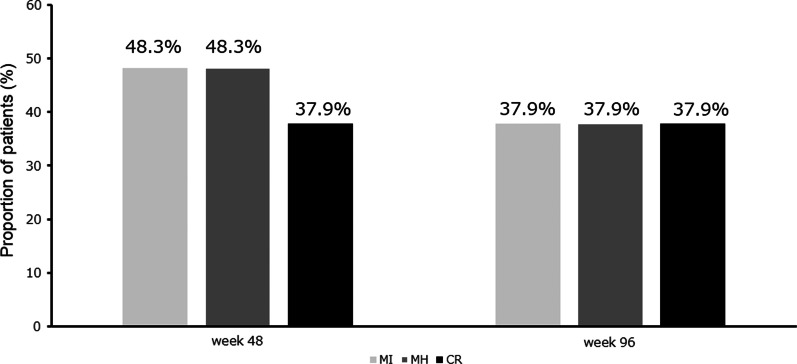
Response rates of anti-TNFα agent (at weeks 48 and 96)

Next, we investigated the response rate of anti-TNFα agents in patients with intestinal BD at the indicated time points (Fig. 3). At week 48, 14 (48.3%), 11 (37.9%), and 14 (48.3%) patients achieved MI, CR, and MH, respectively, whereas 11 (37.9%) patients achieved MI, CR, and MH at week 96.

### Predictors of discontinuation of anti-TNFα agents (Table 3)

We investigated factors associated with the discontinuation of anti-TNFα agents in patients with intestinal BD. To identify predictors of discontinuation of anti-TNFα therapy, we conducted a logistic regression analysis stratified by demographic variables such as sex (male *vs*. female), age at disease onset (≥ 40 vs. < 40 years), disease duration before anti-TNFα agent initiation (≥ 5 vs. < 5 years), type of anti-TNFα agent administered (IFX *vs*. ADA), CRP level at baseline (≥ 1 vs. < 1 mg/dL), and concomitant use of systemic corticosteroids at a dose of ≥ 20 mg (yes vs. no). The results are presented in Table 3. Univariate analysis showed no significant differences between the two groups in terms of sex, age at disease onset, disease duration before anti-TNFα agent initiation, type of anti-TNFα agent administered, and concomitant use of corticosteroids (≥ 20 mg). Notably, only the baseline CRP level was significantly associated with the discontinuation of anti-TNFα agents at week 48 (*p* = 0.019). In addition, multivariate analysis revealed that only a baseline CRP level of 1 mg/dL or higher was statistically associated with the discontinuation of anti-TNFα therapy at week 48 (*p* = 0.033).

**Table 3:** Univariate and multivariate logistic regression analyses of predictors for discontinuation of anti-TNFα therapy at week 48

	Univariate analysis			Multivariate analysis		
	OR	95% CI	*p* value	OR	95% CI	*p* value
Sex (male vs. female)	4.9	0.79–30.3	0.09	2.7	0.31–22.7	0.37
Age at disease onset (≥ 40 vs. < 40 years old)	2.0	0.38–10.5	0.41	1.9	0.20–17.5	0.59
Disease duration before anti-TNFα agent initiation (≥ 5 vs. < 5 years)	0.4	0.07–2.7	0.38	0.4	0.04–4.96	0.49
Type of anti-TNFα agent administered (IFX vs. ADA)	0.8	0.15–3.8	0.73			
CRP level at baseline (≥ 1 vs. < 1 mg/dL)	9.6	1.45–63.5	0.019	10.5	1.21–90.2	0.033
Concomitant use of systemic corticosteroid (≥ 20 mg) (yes vs. no)	1.5	0.27–8.3	0.64			

ADA, adalimumab; CI, confidence interval; CRP, C-reactive protein; IFX, infliximab; OR, odds ratio; TNFα, tumor necrosis factor α

## Discussion

BD, first described in 1937 by the Turkish dermatologist Hulusi Behcet [16], is a multisystem immune-mediated inflammatory disorder characterized by recurrent oral aphthous ulcers, uveitis, skin lesions, and genital ulcers. In addition, BD may involve the intestinal tract (intestinal BD), nervous system, and vascular system, which is categorized as special-type BD in Japan. Those with special-type BD tend to have a poor prognosis, typically necessitating treatment with potent immunosuppressive agents, such as corticosteroids and immunomodulators. Intestinal BD can also result in complications such as perforations, fistulas, or massive intestinal bleeding, eventually requiring surgical treatment [4].

The pathogenesis of BD remains unclear, and some studies have implicated genetic factors, environmental factors, and subsequent immune abnormalities. Genome-wide association studies have identified several genes associated with susceptibility to BD, including *IL23R-IL12RB2*, *IL-10* [17, 18], and *STAT4* [19]. Interleukin (IL)12 induces Th1 immune responses [20], which leads to the production of Th1 cytokines such as interferon (IFN)γ, TNFα, and IL2. STAT4 is a transcription factor required for the Th1 immune response [21]. Meanwhile, IL23 plays an important role in driving aberrant Th17 immune response [22]. Additionally, Th17 cells also produce several cytokines, including IL17 and TNFα [23]. These results suggesting that TNFα plays a crucial role in the pathogenesis of BD. It has been reported that Serum levels of TNFα have been reported to be increased in patients with BD [24], and tissue samples harvested from intestinal BD lesions were found to express TNFα [25].

IFX and ADA are anti-TNFα monoclonal antibodies that have been highly effective against Th1 inflammation. IFX and ADA are reportedly effective against several diseases, including rheumatoid arthritis [26, 27], CD [28, 29], and ulcerative colitis during the active phase [30]. In patients with BD, IFX has been effective in patients with uveitis resistant to combination therapy with AZA, cyclosporine, and corticosteroids [31]. Also, Vallet et al. have reported the efficacy of anti-TNFα agents in severe and/or refractory BD [32]. Furthermore, previous studies have revealed that anti-TNFα agents are also effective for treating intestinal BD [13, 14]. Herein, our study revealed that 48% of patients achieved MI at week 48. In previous studies, the clinical response rates of anti-TNFα agents for intestinal BD varied between 40 and 60% [13, 14, 33, 34]. This was likely due to differences in evaluation criteria and eligible patients. Thus, anti-TNFα agents are effective for intestinal BD therapy, as suggested by previous reports and our study.

In the present study, the CRP level at baseline was significantly associated with the discontinuation of anti-TNFα therapy at week 48 (*p* = 0.019). There are few reports describing the predictors of sustained response in patients with intestinal BD treated with anti-TNFα agents. Some literatures have shown that early achievement of mucosal healing and low CRP level after administration of anti-TNFα agent might be used as predictors of long-term response [35, 36], however, those predictors will be only available after starting the treatment and confirming the well-responsiveness to it. To the best of our knowledge, current study is the first to show that CRP level over 1 mg/dL at baseline is associated with the higher discontinuation rate of anti-TNFα therapy compared with low CRP level in patients with intestinal BD.

Several studies have reported that clinical disease activity correlates with CRP levels in patients with BD. It has been reported that mean CRP levels increase with the number of involved organs [37], and that the CRP level can be correlated with the Behçet's Disease Current Activity Form (BDCAF), which depends on the accurate history of clinical features present during the month prior to the date of assessment [38]. These results indicate that CRP levels are closely associated with disease activity and response to treatment in patients with BD. CRP is an acute-phase inflammatory protein that is synthesized in the liver upon stimulation by IL6. IL6 is immediately produced by innate immune cells, such as macrophages and monocytes, in response to infections and tissue injuries [39]. Along with transforming growth factor (TGF) β, IL6 induces the differentiation of naïve T cells into Th17 cells, which produce several cytokines, including IL17 [40]. As mentioned above, IL23 plays an important role in the Th17 immune response by promoting Th17 differentiation. Moreover, IL23 is necessary to maintain IL17 production by Th17 cells [41]. IL17, together with IL6, triggers a positive-feedback loop of IL6 expression through the activation of NF-κB and STAT3 in non-immune cells [42]. These mechanisms reportedly play a role in the development of autoimmune diseases such as arthritis, with markedly high serum IL6 levels detected in these disorders [42].

It has been shown that trough levels of anti-TNFα agents reflect the efficacy in rheumatoid arthritis [43] and in CD [44]. Our results indicate that it is important to maintain a trough level of anti-TNFα agents for continued long-term anti-TNFα therapy. CRP levels before treatment are positively associated with TNFα levels [45]; therefore, higher doses of anti-TNFα agents are needed to control disease activity in patients with high CRP levels at baseline. Moreover, baseline CRP levels can be correlated with the continuation of anti-TNFα agents in patients with rheumatoid arthritis [46] and with a colectomy-free rate in patients with ulcerative colitis in the active phase [47]; for continued long-term maintenance therapy with anti-TNFα, we may need to maintain a high trough level of anti-TNFα agents by employing corticosteroids or immunosuppressive agents in patients with high CRP levels at baseline, higher dosages of anti-TNFα agents, or shorter dosing intervals in patients with escalated activity during anti-TNFα therapy.

Our study had several limitations. First, this was a single-center study, and the sample size was relatively small. Moreover, our study had a retrospective design. Finally, the disease severity evaluated using endoscopic findings before treatment was not included. However, we believe that our data will provide useful insights when clinicians encounter patients with severe intestinal BD.

## Conclusions

The 48-week continuation rate of anti-TNFα agents was 75.9% in bio-naïve patients with intestinal BD. Patients with a CRP level of 1 mg/dL or less at baseline could continue anti-TNFα therapy for a prolonged duration.

## Data Availability

The datasets used and/or analysed during the current study are available from the corresponding author on reasonable request.

## Abbreviations

BD: Behçet's disease
5-ASA: 5-Aminosalicylic acid
AZA: Azathioprine
6-MP: 6-Mercaptopurine
anti-TNFα: Anti-tumor necrosis factor-α
IFX: Infliximab
ADA: Adalimumab
CD: Crohn’s disease
HLA: Human leukocyte antigen
AEs: Adverse events
CG: Continuation group
DG: Discontinuation group
GI: Gastrointestinal
MI: Marked improvement
CR: Complete remission
MH: Mucosal healing
CRP: C-reactive protein
IL: Interleukin
IFN: Interferon
BDCAF: Behçet's Disease Current Activity Form
TGF: Transforming growth factor
